# Gadolinium-doped Au@prussian blue nanoparticles as MR/SERS bimodal agents for dendritic cell activating and tracking

**DOI:** 10.7150/thno.42114

**Published:** 2020-05-15

**Authors:** Cai Zhang, Zhiwen Xu, Huixia Di, Erzao Zeng, Ying Jiang, Dingbin Liu

**Affiliations:** College of Chemistry, Research Center for Analytical Sciences, State Key Laboratory of Medicinal Chemical Biology, Tianjin Key Laboratory of Molecular Recognition and Biosensing, and Collaborative Innovation Center of Chemical Science and Engineering, Nankai University, 94 Weijin Road, Tianjin 300071, China

**Keywords:** dendritic cell, Au@prussian, bimodal agents

## Abstract

*In vivo* tracking of dendritic cell (DC) migration to the lymphatic system is essential for evaluating the outcome of DC-based immunotherapies. Novel multimodal imaging strategies with high analytical performance are urgently needed to supply complementary information about the migration and colonization of DCs. In this study, we designed a bimodal imaging agent, namely Au@Prussian blue-Gd@ovalbumin nanoparticles (APG@OVA NPs), for activating DCs and real-time tracking of DC migration process by magnetic resonance imaging (MRI). Moreover, the distribution of the colonized DCs in the lymphatic system was profiled at the single-cell levels based on surface-enhanced Raman scattering (SERS) technique.

**Methods**: In this strategy, PBs as cyanide (CN)-bridged coordination blocks were assembled onto the gold nanoparticles core to provide SERS signal in the Raman-silent region (1800 and 2800 cm^-1^), which could avoid background signal interference. The doping Gd^3+^ located in the lattice of PB enables the MRI ability with high relaxivity of the probe. Ovalbumin, an egg allergen, was used as an antigen to activate DCs due to its immunological properties. The prepared APG@OVA NP agents were used to activate DCs with high efficacy and to track their migration and distribution *in vivo* through SERS/MR bimodal imaging.

**Results**: The APG@OVA NP agents could not only enable DC activating and labeling, but also achieve real-time monitoring of DC migration *in vivo* and accurate profiling of DC distribution in the lymphatic system. MR imaging indicated the time-dependent migration of the APG@OVA NP-labeled DCs from the footpad to the sentinel lymph node. The background-free Raman mapping of the lymph node tissue slice demonstrated that the activated DCs have successfully colonized to the sentinel lymph node.

**Conclusion**: Concerning the high activating efficacy, dual complementary imaging readouts, and low biological toxicity, the APG@OVA NPs act as high-performance tracking agents for DC-based immunotherapies.

## Introduction

Dendritic cells (DCs) as the dedicated antigen-presenting cells are instrumental in the initiation of adaptive immunity by presenting antigen to lymph nodes 1. Recently, DC-based tumor immunotherapy has invoked great interest due to the high potential in the treatment of cancer 2. Activated DCs effectively transfer to the lymphatic system is required for effective antitumor therapies, because the colonized DCs in the sentinel lymph node could present antigenic information to T cells to activate specific immunity 3. Monitoring of DCs migration is thus essential for accurate assessment of the efficiency of DC-based immunotherapies.

Up to now, various imaging strategies have been developed to track DCs, including optical detection 4, magnetic resonance imaging (MRI) 5, single-photon emission computed tomography 6, and positron emission tomography imaging methods 7. Among these techniques, MRI is one of the most promising approaches for *in vivo* DC tracking due to its unique advantages, such as widespread use in preclinical and clinical imaging, no radiation hazard, high spatial resolution, excellent soft tissue imaging capability and no depth limitation 8. However, single imaging strategies have great difficulties in offering comprehensive information and possessing high performance with sufficient resolution, sensitivity, and availability 9-12. Multimodal imaging is capable of combining the advantages of different imaging techniques, thus providing multi-dimensional information for an in-depth understanding of the biological process, such as DC migration and colonization.

Compared to the traditional optical imaging methods, surface-enhanced Raman scattering (SERS) shows unique advantages, like insusceptibility to photobleaching and unmatched detection sensitivity 13-17. More importantly, SERS imaging in the Raman-silent region (1800 and 2800 cm^-1^) could avoid background signal interference because biological samples do not possess any Raman signals in this region 18. At present, SERS has been integrated with other complementary imaging methods (i.e., MRI, photoacoustic imaging, fluorescence imaging, and computed tomography) for the diagnosis of lesions, cell tracking, and imaging-guided treatment of various diseases 19-22. Recently, we have developed Prussian blue (PB)-coated gold nanoparticles (AuNPs) as highly sensitive and background-free SERS reporters, which have shown great application potential for molecular detection and imaging 23. On the other hand, PB, also called iron(III) hexacyanoferrate(II), is composed of two different iron centers Fe^3+^ and Fe^2+^ bridged by the CN groups, which has a face-centered cubic structure 24-27. In the crystal structure of KFe^III^[Fe^II^(CN)_6_]·nH_2_O (n = 14-16), the most common form of PB, Gd^3+^ could replace the interstitial positions occupied by K^+^ to generate the Gd^3+^-dopped PB NPs 24. After incorporating with Gd^3+^, the PB probes with high relaxivity and biocompatibility have been successfully used in biosensing and theranostics 28-32. The Gd^3+^-doped PB crystals, however, have not been employed as a sensitive and low-background MRI/SERS multimodal imaging probe for biomedical uses, particularly those coupled with therapeutic agents.

**Scheme 1:**
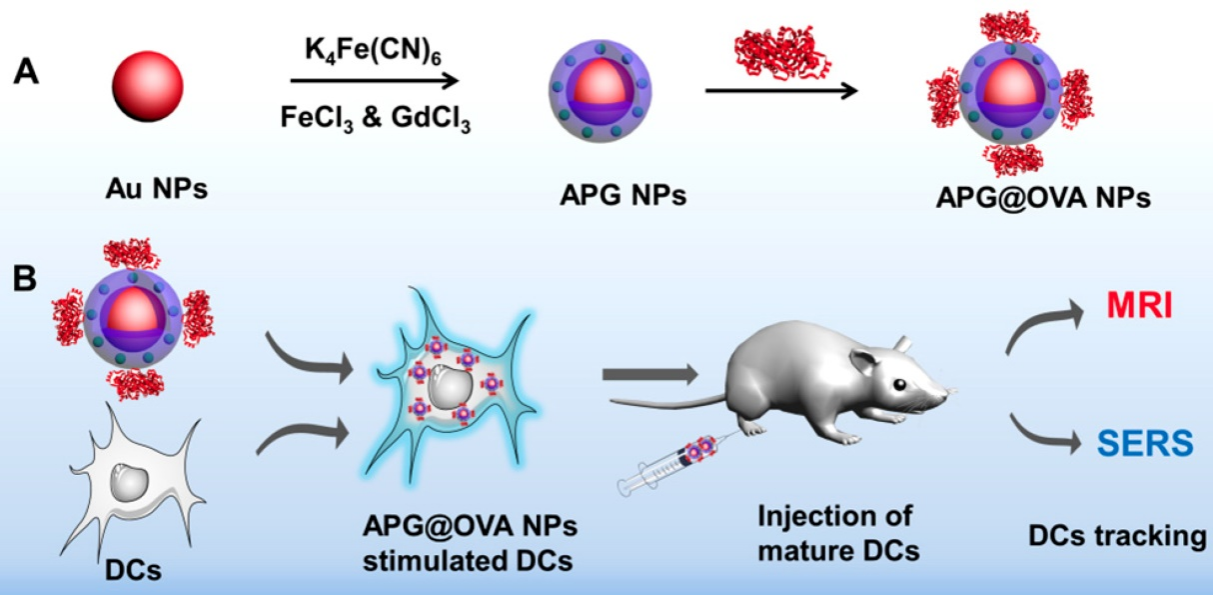
(A) Fabrication process of the APG@OVA NPs and (B) schematic illustration of the APG@OVA NP-based dendritic cells activating and SERS/MR bimodal tracking.

Herein, we developed a multi-functional Au@PB-Gd@OVA nanoparticles (APG@OVA NPs) (**Table S1**) agent for DCs activating and tracking *in vivo*. In this agent, PB as cyanide (CN)-bridged coordination polymer was assembled onto the AuNP core to provide a strong yet background-free SERS signal (**Scheme 1**). The doped Gd^3+^ endows highly sensitive MR imaging ability without influencing Raman intensity. The further coating of ovalbumin (OVA) model antigen through physical absorption endows the particles with the capability of DC stimulating. The fabricated APG@OVA NPs exhibited uniform core-shell nanostructure, excellent biocompatibility, background-free Raman signal, and ultrahigh T1 relaxivity, holding great promises for theranostic applications.

## Experimental section

### Chemicals and instruments

Sodium citrate, tetrachloroauric acid (HAuCl_4_), ferric chloride hexahydrate potassium hexacyanoferrate(II) trihydrate (K_4_[Fe(CN)_6_]·3H_2_O), (FeCl_3_·6H2O), ovalbumin (OVA) and 3-(4,5-dimethyl-2-thiazolyl)-2,5-diphenyl-2-H-tetrazolium bromide (MTT) were obtained from Sigma-Aldrich. DMEM and fetal bovine serum (FBS) were obtained from GIBCO.

Field emission transmission electron microscopy (TEM) (Hitachi, HT7700) was used to study the morphology nanoparticles. The core-shell structure and elemental compositions were analyzed by HAADF-STEM imaging and EDX elemental mapping (Philips Tecnai G2 F20). UV-vis-NIR absorption spectra were recorded thorough U-3900 spectrophotometer (Hitachi). SERS detection was carried out with a Renishaw micro-Raman system including a research-grade Leica DMLM microscope. Dynamic light scattering (DLS) was tested on a Malvern Zetasizer (Nano series ZS, UK). Powder X-ray diffraction (XRD) was recorded by the X-Ray Powder Diffractometer (BrukerD8FOCUS). IR spectra were collected by a FT-IR Spectrometer (Bio-rad, FTS6000). MTT results were detected by a microplate reader (BioTek, Synergy S32/SLFPAD).

### Preparation of Au NPs

The Au NPs with a diameter of 40 nm were prepared by the classic sodium citrate reduction method. Briefly, 5 mL of sodium citrate solution (0.5% w/v) was added into 295.5 mL of boiling HAuCl_4_ solution (0.25 mM) under vigorous stirring at 125°C. The solution was kept boiling for 15 min after it turned to dark red.

### Preparation of APG NPs

K_4_[Fe(CN)_6_] and FeCl_3_ were used as the PB precursors, and GdCl_3_ was used as the gadolinium source to fabricate the APG NPs. To obtain a uniform core-shell structure of the AP NPs, the thickness of PB shell in the AP NPs was optimized by regulating the adding amounts of the PB precursors (K_4_[Fe(CN)_6_] and FeCl_3_). Subsequently, in order to obtain the MR imaging ability, Gd^3+^ was doped into the PB shells by using the mixture of FeCl_3_ and GdCl_3_ instead of FeCl_3_ to generate APG NPs. Briefly, aqueous FeCl_3_ solution was mixed with GdCl_3_ solution at a molar ratio of 5:2, with the final concentration of Fe^3+^ and Gd^3+^ were 5 mM and 2 mM, respectively. 50 mL of the prepared Au NPs (0.3 nM) was put into a round-bottomed flask. Subsequently, 430 μL of 5 mM K_4_[Fe(CN)_6_] solution and an equal volume of the mixed solution of FeCl_3_ & GdCl_3_ were simultaneously added into the Au NPs under vigorous stirring, and such processes need to be repeated for 10 times. Then the mixed solution was vigorously stirred for 3 h. The obtained APG NPs were purified by centrifugation (8000 rpm for 10 min) and washed with water for 2 times.

### Preparation of APG@OVA NPs

10 mL of the as-prepared APG NPs (0.3 mg/mL) was dropwise added into 10 mL of aqueous OVA solution (5 mg/mL) under gently stirring and then the mixture was kept at 37 °C overnight. The obtained APG@OVA NPs were purified by centrifugation (8000 rpm for 10 min) and washed by water for 2 times. Finally, the purified APG@OVA NPs were stored at 4°C.

### Loading Capacity and Stability of APG@OVA NPs

The loading capacity of OVA on APG NPs was detected by the bicinchoninic acid (BCA) protein assay (Beyotime) following the vendor's instructions. To investigate the stability of OVA on APG NPs, APG@OVA NPs were dissolved in different buffer solutions (10 mM, pH 7.4 or pH 5.5) and then kept for various time. After centrifugation (14800 r / 5 min), the released OVA was measured by the BCA protein assay. Furthermore, the colloidal stability of the APG@OVA NPs in different media was also determined by obtaining the Raman signals of APG@OVA NPs in different media (GSH, RPMI-1640, PBS, BSA, and FBS).

### T1-Weighted MRI Properties of APG NPs

APG and APG@OVA solutions with varying concentrations of Gd^3+^ were prepared for *T_1_* relaxivity measurements and *T_1_*-weighted MR images at 25 °C using a 0.5 T small MR imaging system (MesoMR60, Shanghai Niumag Corporation, China). The *T_1_* relaxivity and *T_1_*-weighted MR images were obtained with the parameters: multi spin-echo, TR/TE = 2000/60 ms, slices = 1.

### Generation of Murine Bone Marrow-derived DCs (BMDCs)

Bone-marrow cells were generated according to the previous method. Simply, the femur and tibiae were harvested from the C57BL/6 mice, after that, the bone marrow was flushed out by the RPMI-1640 culture medium. The RBCs were depleted using RBC lysis buffer (Sigma Chemical Co.), and the remaining cells were cultured in an RPMI-1640 culture medium that contains 10% FBS, 1% of streptomycin-penicillin and glutamine, and 25 ng/ml of GM-CSF at 37 °C in 5% CO_2_ environment. The culture medium was changed on the 3^rd^ day, and the cells were collected on the 6^th^ day.

### Toxicity Study of APG@OVA NPs *In Vitro* and* In Vivo*

Three types of cell lines (DC2.4, 3T3, BMDC) were used for investigating the cytotoxicity of APG@OVA NPs. The cells were cultured in 96-well plates (10^4^ cells per well) for 12 h. Then the cell supernatants were substituted with the fresh medium which contained different concentrations of APG@OVA NPs or PBS, and the cells were cultured at 37 °C for another 24 h. After incubation, the cell samples were washed with PBS, and then the fresh medium with 10 μL of 5 mg/mL MTT was added to each well. 4 h later, the cell supernatant was removed and 120 mL of DMSO was added into each well to dissolve the formazan crystal. Finally, the absorbance of the samples at 570 nm was recorded by a microplate reader (Bio-tek). As the control, the viability of the cells treated with PBS was assigned as 100%, base on which the relative viability of cells treated with APG@OVA NPs was determined. Subsequently, the in vivo toxicity of the APG@OVA NPs was investigated by the histopathological test of the major organs from the experimental mice. Briefly, the major organs (including liver, heart, lung, spleen, and kidney) of the mice that treated with the APG@OVA NPs labeled DCs were collected after injection for 1 and 7 days. The mice without any treatment were used as control.

### Cytokine Detection

10^4^ of BMDCs were cultured in 96-well plates overnight, and APG@OVA NPs with the final concentration of 50 mg/L were mixed the cells and cultured for 24 h. Meanwhile, OVA (10 mg/L, the same concentration in 50 mg/L APG@OVA NPs) and APG (40 mg/L) were cultured with BMDCs for 24 h. The cells without the treatment of APG@OVA NPs were used as control. Afterward, the suspension of the cell samples was diluted for analysis. Interleukin-6 (IL-6) and tumor necrosis factor α (TNF-α,) were tested by the cytokine-specific ELISA kits, and all the samples were measured in triplicate.

### Flow Cytometry (FACS) Assay

For further investigating the maturation level of BMDC after treated with APG@OVA NPs, the expression levels of surface molecules (CD 40, CD 80, CD83, CD86, and MHC-II) were evaluated by FACS. After being treated with APG@OVA NPs, BMDCs were washed with PBS and stained with anti-CD40-FITC (BD Pharmingen), anti-CD80-FITC, anti-CD83-PE, anti-CD86-PE and anti-MHC-II-FITC antibodies (MultiSciences) for 20 min at room temperature. The cellular fluorescence of these BMDCs was measured by FACS analysis (BD FACS) after being washed with PBS.

### *In Vitro* T cell Activation

Mouse spleen lymphocytes were obtained from C57BL/6 mice. BMDCs were treated with APG@OVA (50 mg/L) or RPMI-1640 medium for 5 h. After that, the spleen lymphocytes were co-incubated with RPMI-1640 medium or the treated DCs at a ratio of 3: 1 for 48 h, respectively. Finally, the supernatants of these cell samples were collected and centrifuged for IFN-γ analysis. IFN-γ secretion was evaluated by the mouse IFN-γ ELISA kit (MultiSciences Co. Ltd., Hangzhou, China) according to the manufacturer's protocols.

### Raman Imaging of Labeled DCs

DCs were plated onto glass slides in 24-well plates at a concentration of 10^4^ per well and cultured at 37°C overnight. Then APG@OVA NPs (50 mg/L) were added to the cell samples and incubated for different time to optimize the optimal incubation time (8, 24, 48, 72, and 96 h). After the cell supernatants being removed, the cells were washed with PBS five times and fixated with 4% paraformaldehyde. The Raman mapping of the BMDCs that treated with APG@OVA NPs was recorded by a Raman confocal microscope with a 633 nm laser, with a 50× objective lens, a power of 3 mW, and an exposure time of 1 s.

### Labeling and MR Imaging of DCs *In Vitro*

To investigate the in vitro DCs labeling sensitivity of the APG@OVA NPs, the probes were incubated with DCs for 24 h. After being washed 3 times with PBS, the APG@OVA NPs labeled-DCs were collected and diluted in DMEM at a graded cell number (5 x 10^4^, 5 x 10^5,^ and 2 x 10^6^ cells). *In vitro* MR imaging ability of the labeled DCs was performed under a clinical MRI scanner (3.0 T).

### MRI/SERS Bimodal Tracking of Labeled DCs* In Vivo*

BMDCs were incubated with APG@OVA NPs for 24 h, then collected and resuspended in 1640 medium. Afterward, the cell samples (2 x 10^6^ cells) were injected into C57BL/6 mice at the left hind leg footpads. After injection, MRI scanning was carried out under a 3.0 T clinical MRI scanner at different time points (0, 24, 48 and 72 h) to monitor the DCs migration process, with *T_1_*-weighted fast spin-echo sequence (FA = 111°; TR/TE = 360/10 ms; slice thickness = 1 mm without gap). Then the mice were sacrificed post-injection of the APG@OVA NPs labeled-DCs for 72 h and the left inguinal lymph nodes of mice were cut out. Subsequently, lymph nodes were sliced for Raman mapping, and immunohistochemical staining (anti-CD11c (Servicebio) was used to stain DCs in the lymph nodes). Raman mapping was performed by a Raman confocal microscope with a 633 nm laser, a power of 3 mW, a 50× objective lens, and the exposure time was 1 s.

To assess the distribution of the labeled-BMDCs in different organs in vivo, APG@OVA NP-labeled BMDCs (2 × 10^6^ cells / 50 µL) were intravenously injected into C57BL/6 mice to follow and detect BMDCs. After injection, MRI scanning was carried out under a 3.0 T clinical MRI scanner at different time points (1, 3, 4, and 24 h) to monitor the DCs.

## Results and Discussion

### Preparation and Characterization of APG@OVA NPs

To obtain the APG@OVA NPs, Au NPs were firstly fabricated as the cores and then coated with Gd^3+^-doped PB shells and OVA model antigen in sequence 33. In detail, the Au NPs were fabricated through a classic sodium citrate reduction method. Then the Gd^3+^-doped PB shells were synthesized following our previous method with some modification 23. To obtain a uniform core-shell structure, the morphology of PB shells in AP NPs was optimized by adding different amounts of precursors (K_4_[Fe(CN)_6_] and FeCl_3_). **Figure S1** showed that the PB shells around the Au NP cores were patchy at low concentration of the precursors. The thickness of the PB shell increased as the amounts of precursors increased (0.13, 0.27, 0.4, and 0.51 mM of K_4_[Fe(CN)_6_] and FeCl_3_ ). When the concentration of the precursors reached 0.4 mM, the PB shells became regular core-shell nanostructures. The SERS intensity of the probes increased as the PB shells becoming thicken, with the color changed from purple to dark blue (**Figure S2**). In order to obtain the MR imaging ability, Gd^3+^ was doped into the PB shells by using the mixture of FeCl_3_ and GdCl_3_ instead of FeCl_3_ alone to generate APG NPs. The OVA proteins were coated to the APG NPs through the inherent absorption capacity of proteins to nanoparticles.

**Figure 1:**
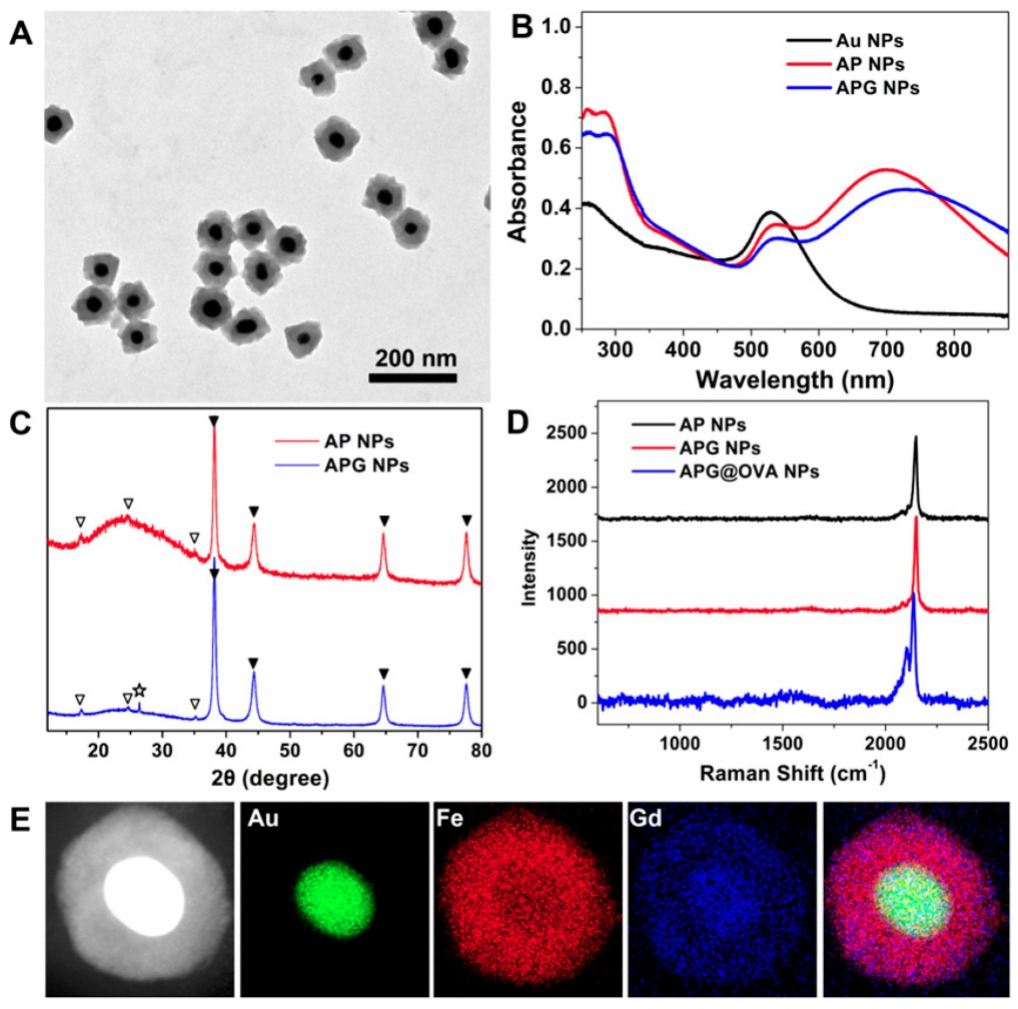
(A) TEM image of APG NPs. (B) UV-vis-NIR absorption spectra of Au NPs, AP NPs, and APG NPs. (C) XRD patterns of AP NPs and APG NPs. Two series of peaks are assigned to the Au core (marked with solid triangles) and the PB (marked with hollow triangles). The pentangle represents the signal from the gadolinium hexacyanoferrate. (D) Raman spectra of AP NPs, APG NPs, APG@OVA NPs that collected by a confocal Raman spectrometer using 633 nm (3 mW) laser excitation. Data acquirement time, 0.1 s. (E) HAADF-STEM image of APG NPs and the EDX elemental mapping of the Au core, PB shell, doped Gd^3+,^ and the merged image.

Transmission electronic microscopy (TEM) images demonstrated that the as-prepared APG NPs possessed monodisperse uniform core-shell nanostructures (**Figure 1A**). The obtained NPs were then characterized by UV-vis-NIR spectra, powder X-ray diffraction (XRD) patterns, DLS, and infrared spectra. The AP NPs possessed two absorption peaks at 532 and 700 nm (**Figure 1B**), which are responded to Au NPs and PB NPs, respectively. After Gd^3+^ doping, the PB absorption peak redshift to 728 nm. XRD patterns demonstrated that such AP NPs containing two components: gold structure (JCPDS card no. 89-3697) and PB (JCPDS card no. 1-239). After Gd^3+^ doping, a characteristic peak at 26.4° appears in XRD, which belongs to the diffraction plane for gadolinium, indicating that Gd^3+^ is attached to the cyanide bonds covalently (**Figure 1C**) 34. The infrared spectra (**Figure S3**) of AP NPs showed a single band at about 2084 cm^-1^ in the CN stretching region, indicating the production of CN-bridged polymeric complex around the Au NPs. The bands at 1645 and 1533 cm^-1^ demonstrated the presence of the OVA proteins. The hydrodynamic diameters of APG NPs and APG@OVA NPs were determined to be 123 nm and 220 nm, respectively (**Figure S4**). The Raman spectra showed the three types of NPs possess a sharp and strong Raman band in the Raman-silent region, which is assigned to the stretching vibration of CN in PB (**Figure 1D**). The energy-dispersive X-ray spectroscopy (EDS) mapping images in **Figure 1E** demonstrated the element distribution of the core-shell APG NPs.

### T1-Weighted MRI Properties

**Figure 2:**
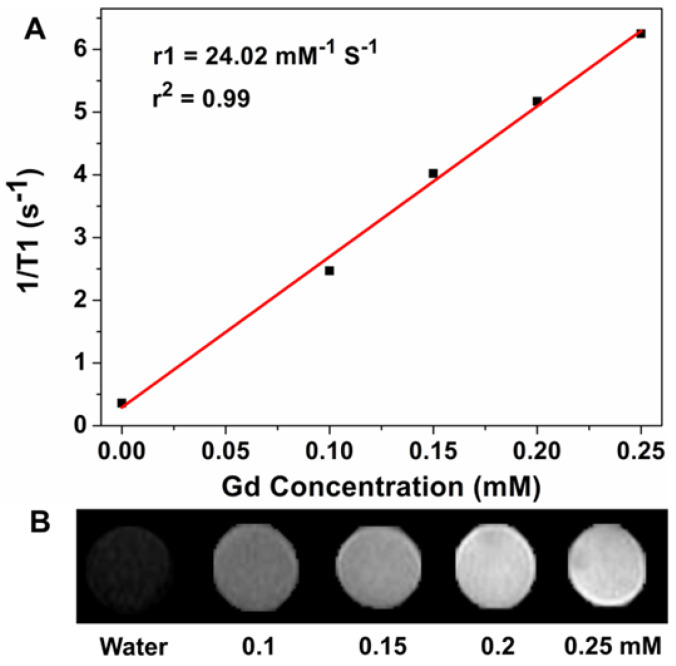
(A) Linear fitting of 1/*T_1_* as a function of Gd^3+^ concentrations at a magnetic field strength of 0.5 T for APG@OVA NPs. (B) Corresponding *T_1_*-weighted MR phantom image of APG@OVA NPs of varied Gd^3+^ concentrations using a 0.5 T MRI scanner.

To evaluate the T1-weighted MR imaging property of the APG and APG@OVA NPs as contrast agents, the relaxivity values *r_1_* of the APG and APG@OVA NPs with a serious of Gd^3+^ concentrations were determined at a 0.5 T small animal MRI system. The *r_1_* value of the APG and APG@OVA NPs was calculated to be 16.5 and 24.02 mM^-1^ s^-1^, respectively (**Figure 2A and Figure S5**). The *T_1_*-weighted MR images of APG@OVA NPs showed a concentration-dependent brightening effect (**Figure 2B**). These results indicated that the APG and APG@OVA NPs possessed excellent *T_1_*-weighted MR imaging ability.

### Loading Capacity and Stability of APG@OVA NPs

The loading capacity of OVA on APG NPs was about 20%, which evaluated by the bicinchoninic acid (BCA) protein assay (Beyotime). The stability of OVA on APG NPs was assessed by dissolving APG@OVA NPs in different buffer solutions (10 mM, pH 7.4 or pH 5.5) and then kept for various time. After centrifugation (14800 r, 5 min), the released OVA was measured by the BCA protein assay (**Figure S6**). Only a few OVA was detected in the supernatants under pH 7.4 after centrifugation, and the released OVA of the APG@OVA NPs in pH 5.5 buffer solutions was less than 20% till co-cultured for 12 h. Furthermore, the colloidal stability of the APG@OVA NPs in different media was also determined by obtaining the Raman signals of APG@OVA NPs in different substrates. The results in **Figure S7** showed that the interference of several substrates to the Raman intensity of APG@OVA NPs is slight.

### In Vitro and In Vivo Toxicity

Three types of cell lines (DC2.4, 3T3, BMDC) were used for investigating the cytotoxicity of the APG@OVA NPs. **Figure S8** revealed over 84% of cells maintained alive after treating with 100 mg/L of the APG@OVA NPs for 24 h. The viabilities of the cell samples were still above 69% even when the concentration of the APG@OVA NPs reached as high as 200 mg/L, 4 folds higher than the optimized concentration (50 mg/L) used in the subsequent DC activation experiments. In order to verify the *in vivo* toxicity of the APG@OVA NPs, the major organs of the mice treated with APG@OVA NP-labeled DCs were collected at the 1^st^ and 7^th^ days for pathological analysis. **Figure S9** revealed that no significant histological changes were observed in the major organs from the mice post-injected with the labeled BMDCs, compared to the control group. These results indicated that the APG@OVA NPs were biocompatible and suitable for biological application.

### Cytokine Detection

**Figure 3:**
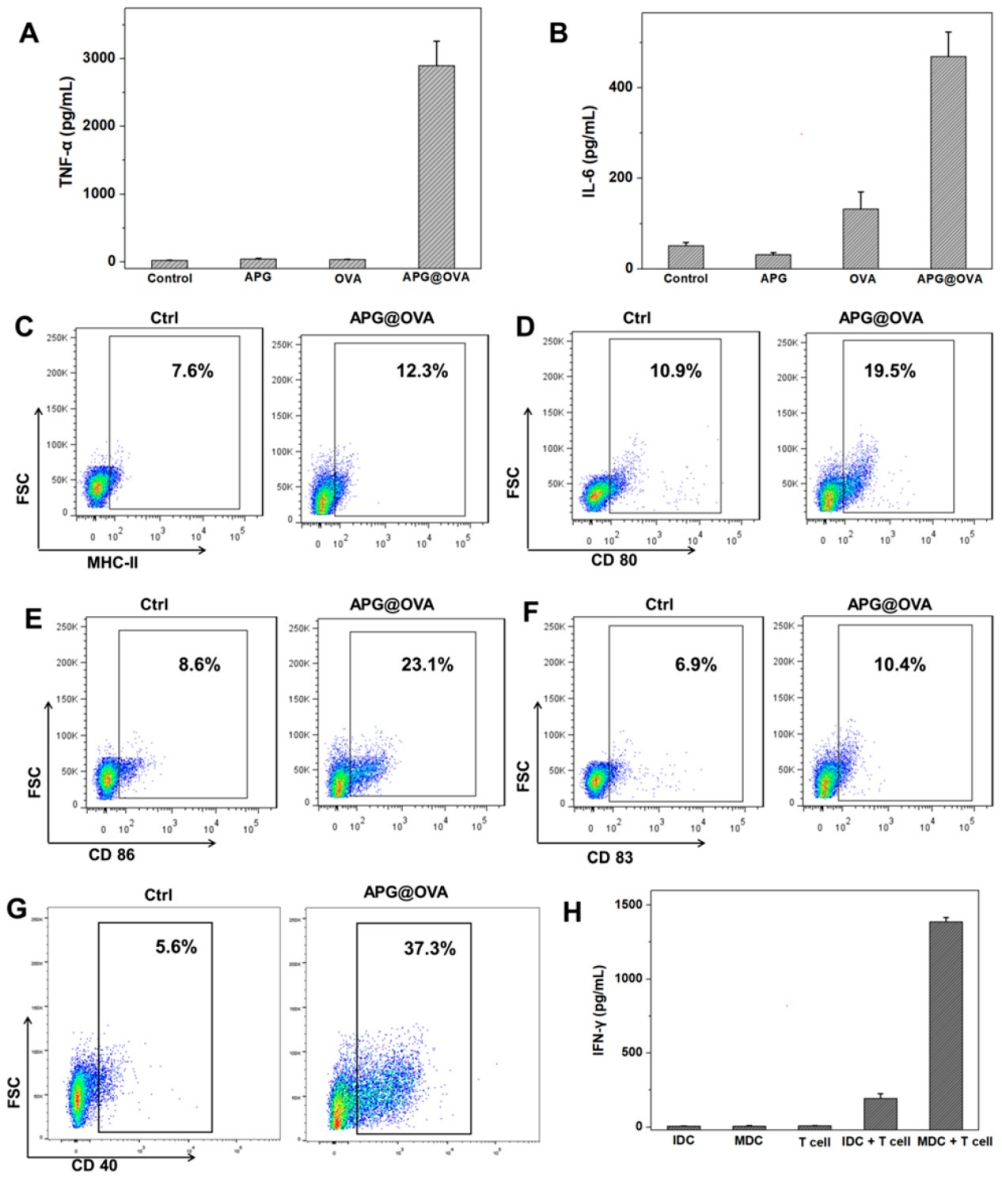
BMDC activation of APG@OVA NPs and *in vitro* T cell activation. The concentrations of TNF-α (A) and IL-6 (B) in the BMDC culture supernatants after activation with APG NPs, pure OVA, and APG@OVA NPs. The BMDCs without any treatment were set as control. Error bars indicate mean standard deviations of six parallel samples for each case. (C-G) FACS quantification of the expressions of MHC-II, CD80, CD86, CD83, and CD40. (H) The cytokine IFN-γ secretion from the T cells incubated with different types of treated BMDCs by ELISA. Error bars represent the mean standard deviations of three replicates.

To evaluate the DC stimulating ability of the APG@OVA NPs, the suspension of BMDC culture media was diluted for analysis after different treatment (PBS, APG, OVA, and APG@OVA). Tumor necrosis factor α (TNF-α) and interleukin-6 (IL-6) were tested with cytokine-specific ELISA kits, and all the samples were measured in triplicate. Compared to the samples treated with either APG NPs or OVA, the levels of IL-6 and TNF-α from the suspension of BMDCs treated with APG@OVA NPs (50 mg/L) showed a significant increase (**Figure 3A-B**). The results demonstrated that the as-prepared APG@OVA NPs had a high efficacy of the enhancement of cytokine release.

### Flow Cytometry (FACS) Assay

MHC-II, CD 40, CD80, CD83, and CD86 are the important surface markers expressed on the DCs, and DC maturation can be evaluated by detecting the upregulation of these molecules on the cellular membrane 35. Antigen-presenting molecules MHC-II can target to T cell receptor and present antigen to T cells providing the “first signal” for T cell stimulation. While costimulatory molecules CD40, CD80, and CD86 can recognize and bind to CD40L and B7 molecules on the T cells, providing the 'second signal' 36. Thus, for investigating the maturation level of BMDC after being treated with APG@OVA NPs, the expression levels of surface molecules (CD 40, CD 80, CD 83, CD86 and MHC-II) of BMDC were evaluated by FACS. As shown in **Figure 3C-G**, the expression levels of surface molecules of the BMDC after being treated with APG@OVA NPs were increased significantly than the control groups. The results demonstrated that the as-prepared APG@OVA NPs can upregulate the expression of surface markers on BMDCs and promotes cell maturation.

### *In Vitro* T Cell Activation

In order to investigate the activation level of T cells after co-culturing with mature BMDCs, IFN-γ secretion of T cells after incubating with different types of BMDCs was evaluated by the mouse IFN-γ ELISA kit. As shown in **Figure 3H**, T cells that co-cultured with mature BMDCs could produce much more IFN-γ than that co-incubated with immature ones. Meanwhile, almost no cytokine IFN-γ was detected in the supernatants of the pure immature BMDCs, mature BMDCs, and T cells. The result suggested that APG@OVA pulsed BMDCs have the ability to present antigen information to T cells and stimulating T cells activation.

### Raman Mapping of Labeled BMDCs

**Figure 4:**
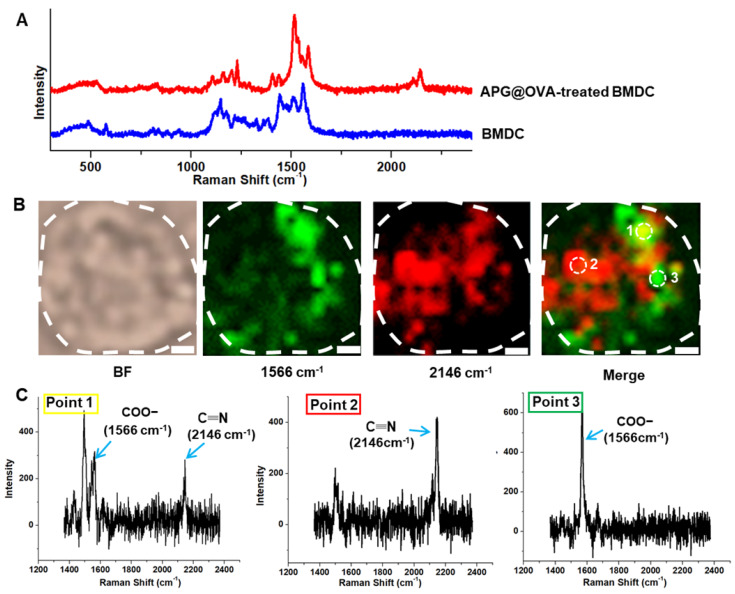
Raman spectra and imaging of BMDCs. (A) Raman spectra of the single BMDC with or without the treatment of the APG@OVA NPs. (B) Bright-field (BF) and Raman mapping images of the BMDC based on the 1566 and 2146 cm^-1^ channels and their merged images. Scale bar: 2 μm. (C) Raman spectra of points 1, 2 and 3.

The APG@OVA NPs possess an intense sharp Raman band in the Raman-silent region due to the mode of CN stretching. Both the doped Gd^3+^ and the coated OVA did not have a noticeable influence on the Raman peak. This property enables the as-prepared APG@OVA NPs as sensitive Raman tags for bioimaging without background noise. To optimize the DC-labeled efficiency of the APG@OVA NPs, 50 mg/L of the probes were incubated with DCs for different time (8, 24, 48, 72, and 96 h). Before Raman imaging, the nonspecific Raman tags were removed by rinsing with PBS three times. **Figure S10** showed that, as the incubation proceeded, the amount of the labeled APG@OVA NPs increased gradually. After incubation for 24 h, the SERS signals reached a maximum and maintained at a high level until 96 h. The spectra of the BMDCs treated with the APG@OVA NPs were recorded by a Raman confocal microscope with a 633 nm laser (3 mW) with the exposure time of 10 s. With respect to the BMDCs without APG@OVA NPs treatment, their spectra were also collected. The Raman spectrum of a single APG@OVA-treated BMDC revealed that complex Raman peaks related to the endogenous cellular components in the fingerprint region, along with a sharp single band (2146 cm^-1^) located in the Raman-silent region (**Figure 4A**). Moreover, the single peak of the APG@OVA NPs was completely separated from the abundant Raman peaks in the fingerprint region arising from endogenous biomolecules of BMDCs.

Traditional Raman reporters like cyanine derivatives and MGITC (malachite green isothiocyanate) possess characteristic peaks in the fingerprint region which would overlap with the complex Raman bands corresponding to the cellular components to produce background noise 37-39. In this regard, the imaging accuracy of the traditional Raman tags would be impacted by the background signals. We developed PB as the Raman dye in which the CN shows a strong and sharp peak at 2146 cm^-1^ in the cellular Raman-silent region that can be easily separated from the background signals. Because biospecies do not show any Raman signals in the silent region, the specific CN signal can accurately reflect the successful labeling of the APG@OVA NPs on the BMDCs. As shown in **Figure 4B**,**C**, The merged image shows that there were abundant overlapping signals (point 1) in green derived from both the Raman tags and cellular components. Nevertheless, the strong CN signal in red (point 2) and the overlaped signals in green can be unambiguously resolved from the background signals. The Raman signals in yellow (point 3) were mainly assigned to the endogenous components (COO-). As confirmed in the Raman spectra, point 1 exhibit both the Raman peaks at 1566 and 2146 cm^-1^. In the spectrum of point 2, only a single peak at 2146 cm^-1^ was presented, without the spectral crosstalk by any other complex Raman signals. The spectrum of point 3 only shows the background signals particularly that for COO-. These results demonstrated that the exogenous CN signal could be used to label the BMDCs reliably and accurately because it can be completely resolved from the background signals. Therefore, Raman mapping of the APG@OVA NPs by means of CN band could facilitate the accurate profiling of BMDCs in complex biological systems with high SBR (signal-to-background ratio).

### Labeling and MR Imaging of DCs *In Vitro*

**Figure 5:**
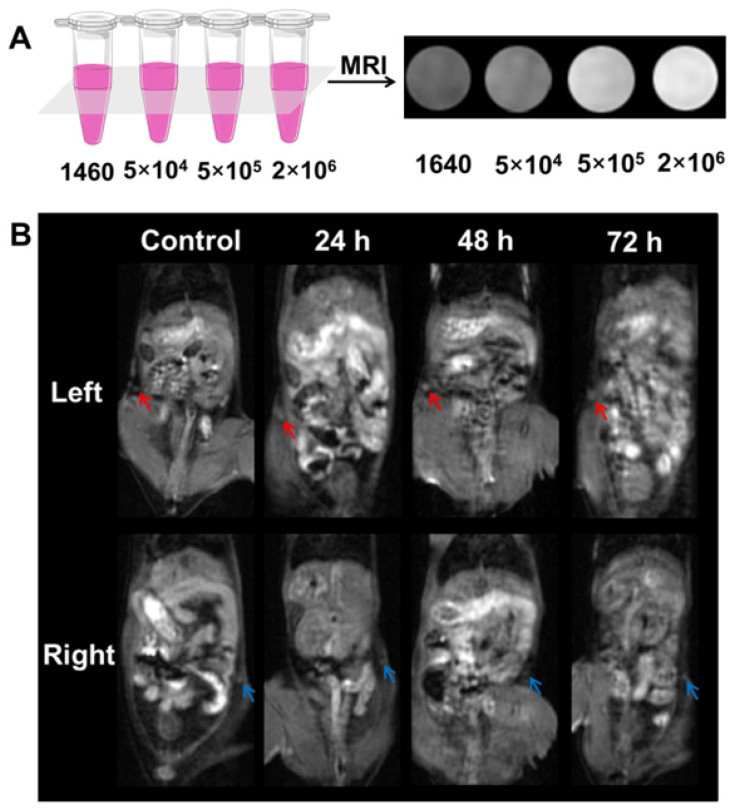
MR imaging of the labeled- BMDCs *in vitro* and *in vivo*. (A) MR imaging of the labeled-DCs *in vitro*. The labeled- BMDCs were diluted in DMEM at graded cell numbers (5 x 10^4^, 5 x 10^5^ and 2 x 10^6^ cells) and the pure DMEM was set as control. (B) MR images of the C57BL/6 mouse that injected with the labeled-BMDCs at left footpads. The left inguinal lymph node (red arrow) was brightening post-injection 24-72 h, while the brightening change of right lymph node (blue arrow) was barely visible.

To investigate MR imaging capability of the labeled-DCs *in vitro*, the DCs were first incubated with the APG@OVA NPs (50 mg/L) for 24 h, and then the labeled-DCs were collected and diluted in 0.05 mL 1640 medium at graded cell numbers (5 × 10^4^, 5 × 10^5^ and 2 × 10^6^ cells). *In vitro* MR signals of labeled BMDCs were detected under a clinical 3.0 T MRI scanner **(Figure 5A)**. The concentration-dependent bright MR images indicated the excellent MR contrast ability of those labeled-BMDCs.

### MRI/SERS Bimodal Tracking of Labeled DCs* In Vivo*

**Figure 6:**
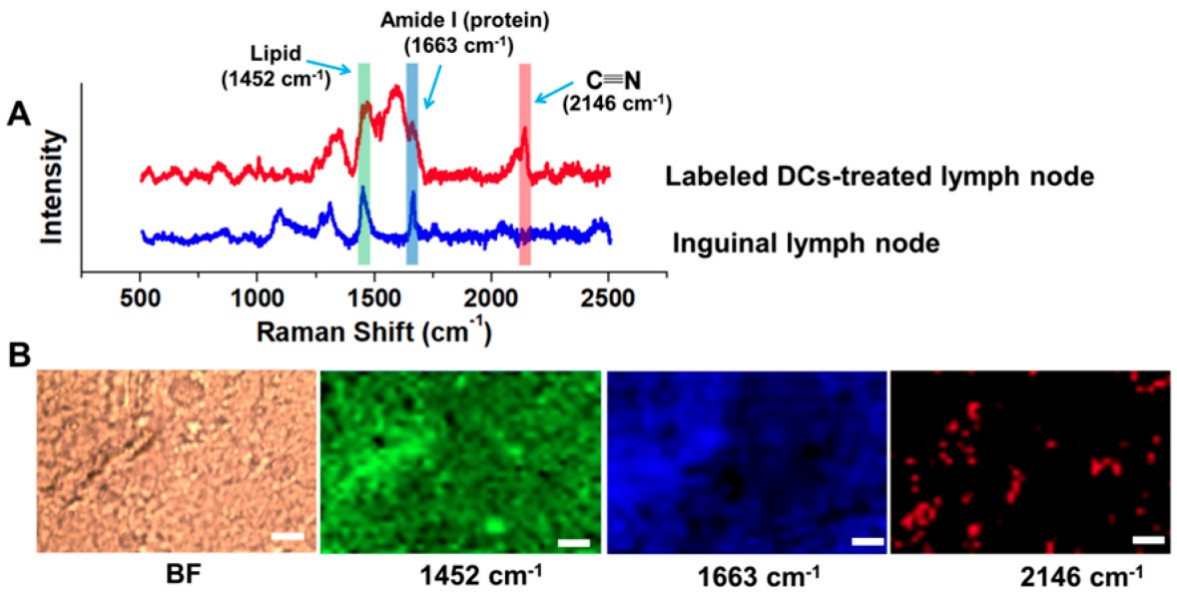
Raman detection and imaging of the inguinal lymph node from the mice treated with APG@OVA-labeled BMDCs. (A) Raman spectra of the inguinal lymph node with or without the APG@OVA-labeled BMDCs. (B) Bright-field (BF), Raman mapping images of the tissue acquired in 1451, 1665, and 2146 cm ^-1^ channels. Scale bar, 20 μm.

To investigate the feasibility of the DC tracking *in vivo*, APG@OVA NP-labeled BMDCs (2 × 10^6^ cells/50 µL) were subcutaneously injected into the left footpads of C57BL/6 mice to monitor the process of DC migration. MR images of the mice were collected at different time intervals under a 3.0 T MR scanner (**Figure 5B**). The inguinal lymph node was brightened at 24 h and became brighter at 48 h after injection of the labeled DCs, which indicated the gradual migration of the APG@OVA labeled DCs from the footpads to the lymph node through lymphatic vessels. For further verifying the migration of the labeled DCs to lymph nodes, the left inguinal lymph node was dissected for SERS detection and immunohistochemical staining. **Figure 6A** showed that the lymph node tissues possess abundant Raman signals in the fingerprint region corresponding to endogenous biomolecules, like lipid, protein, and nucleotide, thus producing strong background interference. While the Raman bands of the CN in the silent region will be completely separated from the background noise to provide the accurate distribution of the APG@OVA labeled DCs. The Raman signals acquired from the 1451 and 1665 cm^-1^ channels were distributed across the tissue, which were assigned to CH functional groups (in lipids, amino acid side chains of the proteins and carbohydrates) and protein respectively. While the peak at 2146 cm^-1^ in the Raman silent region can be resolved entirely from the background signals derived from lymph node tissues. The Raman mapping images of the left lymph node slice possessed reliable CN signals in the Raman-silent region without background interference, demonstrating that the activated BMDCs have successfully migrated and colonized to the sentinel lymph node (**Figure 6B**). The immunohistochemical staining images revealed that the CD11c marker was expressed more in the left lymph nodes than right ones, which indirectly demonstrated the successful migration of BMDCs (**Figure S11**). These results indicated that the exogenous CN signals were more accurate for visualizing the location of the DCs than those appearing in the fingerprint region. Therefore, the proposed APG@OVA NPs could serve as an excellent multifunctional agent for DC activation and tracking *in vivo*.

To assess the distribution of the labeled-BMDCs in different organs in vivo, APG@OVA NP-labeled BMDCs (2 × 10^6^ cells / 50 µL) were intravenously injected into C57BL/6 mice to follow and detect BMDCs. As shown in **Figure S12,** the *T_1_* signal of the vessels was significantly increased at 1, 3 h post-injection of the labeled-BMDCs, and the* T_1_* signal of spleen was enhanced slightly revealed that part of the labeled-BMDCs gathered in the spleen after intravenous injection.

## Conclusions

In summary, we report a multifunctional agent (APG@OVA NPs) for DC stimulating, labeling, and tracking *in vivo*. The exposed OVA molecules on the APG@Ps showed high efficacy in DC activation before subcutaneous injection, while the Gd^3+^-doped PB shells provided background-free SERS signal and sensitive MR signals simultaneously. *In vivo* DC tracking study revealed that the APG@OVA NPs could not only be used for real-time tracking of the DC migration by MR imaging but also reported the reliable distribution information about the labeled DCs colonized at the lymph nodes. Overall, the MRI/SERS-based platform opens up a promising means for DC activating and tracking, which should facilitate the study of DC-based immunotherapies.

## Supplementary Material

Supplementary figures and tables.
