# Supplementary material for: Gadolinium-doped Au@prussian blue nanoparticles as MR/SERS bimodal agents for dendritic cell activating and tracking
Source: Theranostics. 2020 May 15;10(13):6061–71. doi: 10.7150/thno.42114 (PMC7255006; doi:10.7150/thno.42114)
Supplement: Supplementary file 1 — Supplementary figures and tables. [file thnov10p6061s1.pdf]

Supporting Information for

Gadolinium-doped Au@prussian blue nanoparticles as MR/SERS bimodal agents for dendritic cell activating and tracking

Cai Zhang, Zhiwen Xu, Huixia Di, Erzao, Zeng, Ying Jiang, and Dingbin Liu*

College of Chemistry, Research Center for Analytical Sciences, State Key Laboratory of Medicinal Chemical Biology, and Tianjin Key Laboratory of Molecular Recognition and Biosensing, Nankai University, Tianjin 300071, China

AuNPs: gold nanoparticles

AP NPs: Au@Prussian blue nanoparticles

APG NPs: Au@Prussian blue-Gd nanoparticles

APG@OVA NPs: Au@Prussian blue-Gd@ovalbumin nanoparticles

Table S1. Description of abbreviations

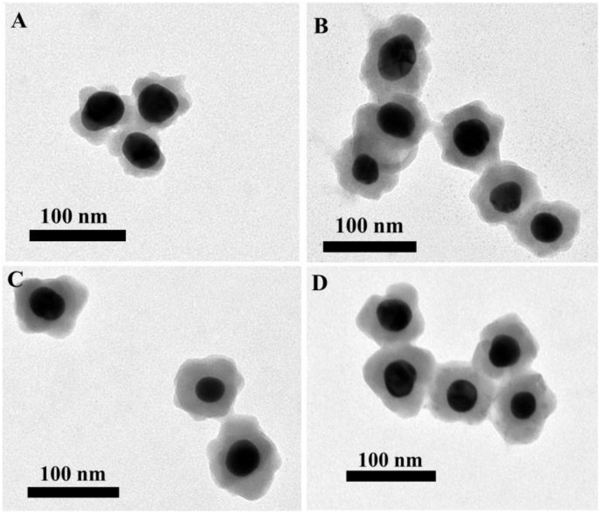

Figure S1. TEM images of AP NPs with various shell thicknesses fabricated by adding different amounts of $K_4[Fe(CN)_6]$ and $FeCl_3$. The thickness of PB shell increased with the addition of PB precursors, A) 0.13 mM, B) 0.27 mM, C) 0.4 mM, and D) 0.51mM.

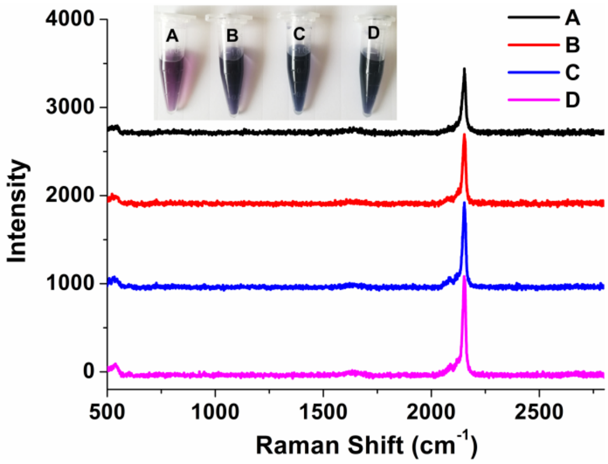

Figure S2. Raman spectra and photos of AP NPs with various shell thicknesses prepared by adding different amounts of PB precursors (A-D corresponding to 0.13, 0.27, 0.4 and 0.51 mM of $K_4[Fe(CN)_6]$ and $FeCl_3$). All the spectra were collected by a confocal Raman spectrometer using 633 nm (3 mW) laser excitation. Data acquirement time, 0.1 s.

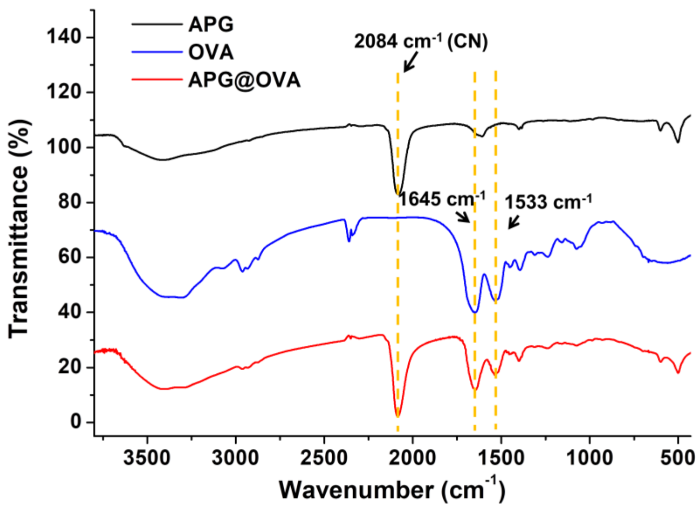

Figure S3. Infrared spectra of APG NPs, OVA, and APG@OVA NPs. The bands at approximately 2084 cm^{-1} are characteristics of CN stretching. Two strong peaks at approximately 1645 cm^{-1} and 1533 cm^{-1} were characteristic of amides I and II, respectively.

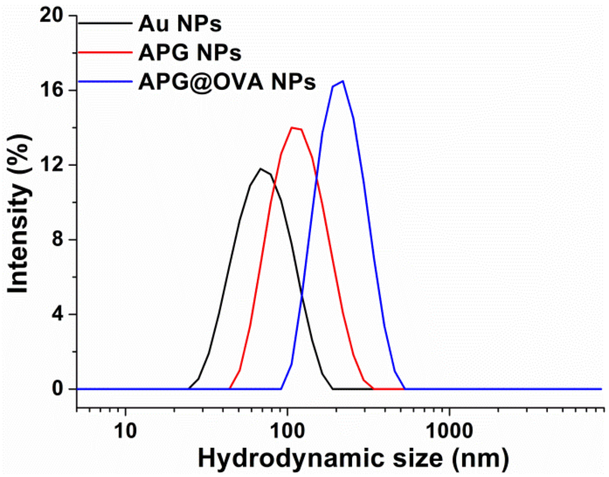

Figure S4. Dynamic light scattering (DLS) data of Au NPs, APG NPs and APG@OVA NPs.

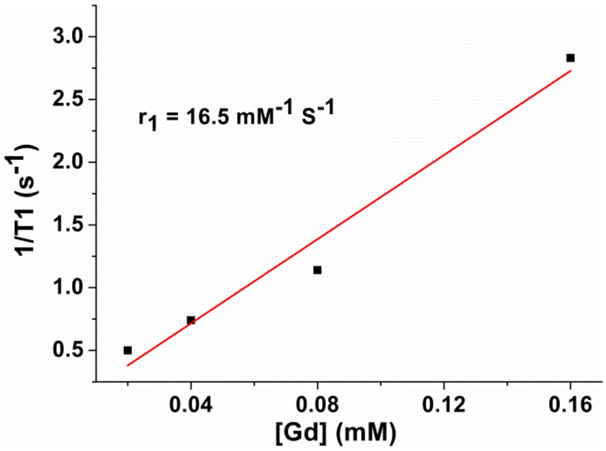

Figure S5. Linear fitting of $1/T_1$ vs Gd^{3+} concentrations at a magnetic field strength of 0.5 T for APG NPs

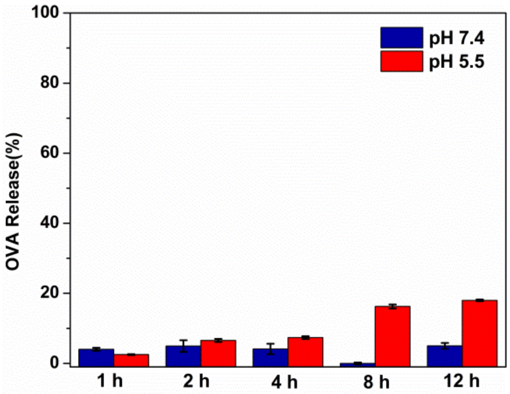

Figure S6. OVA release from APG@OVA NPs under different pH values.

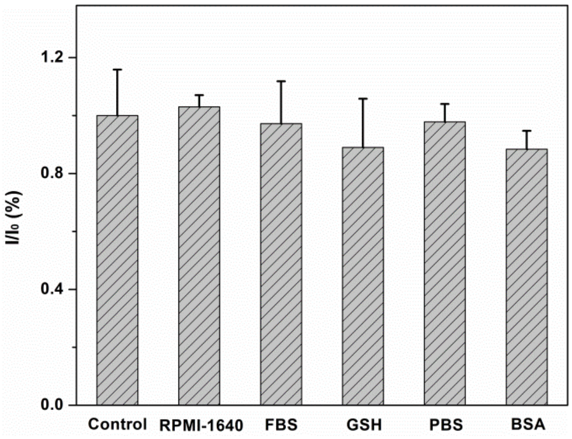

Figure S7. Colloidal stability of the APG@OVA NPs in different media. The final concentration of GSH and BSA was 2 mg/mL. The pure APG@OVA NPs solutions were used as the control. The vertical axis (I/I_0) represents the ratio of the Raman intensity of the mixtures to that of the pure APG@OVA NPs. Error bars represent three sets of repeats.

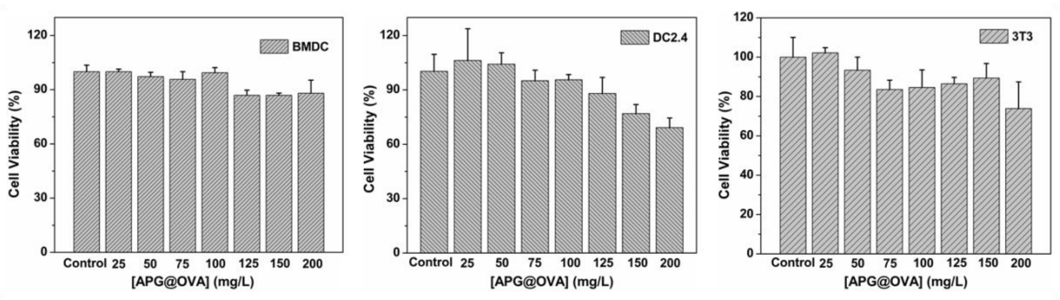

Figure S8. Cytotoxicity APG-OVA NPs evaluated by three cell lines (BMDC, DC2.4 and 3T3 cells) that determined by the classic MTT assays.

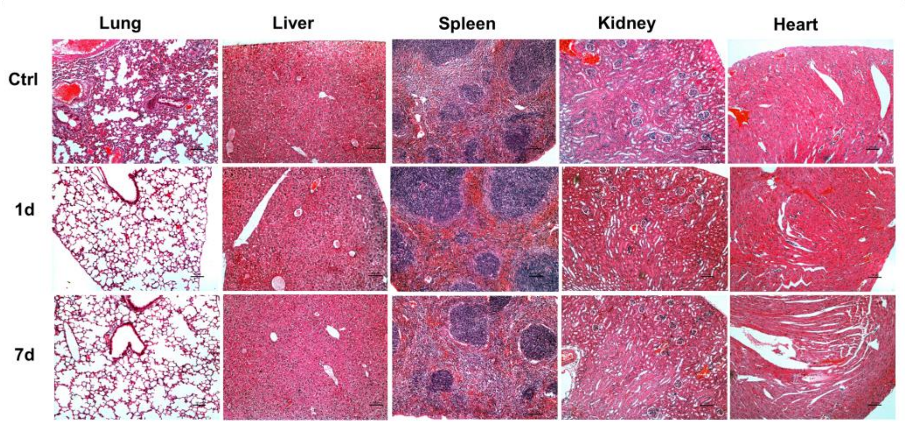

Figure S9. Histopathological results of different organs from the mice after subcutaneous injection of APG@OVA labeled-BMDCs in the foodpads for 1 and 7 days. The mice without any treatment were used as the control. Scale bar, 100 μm.

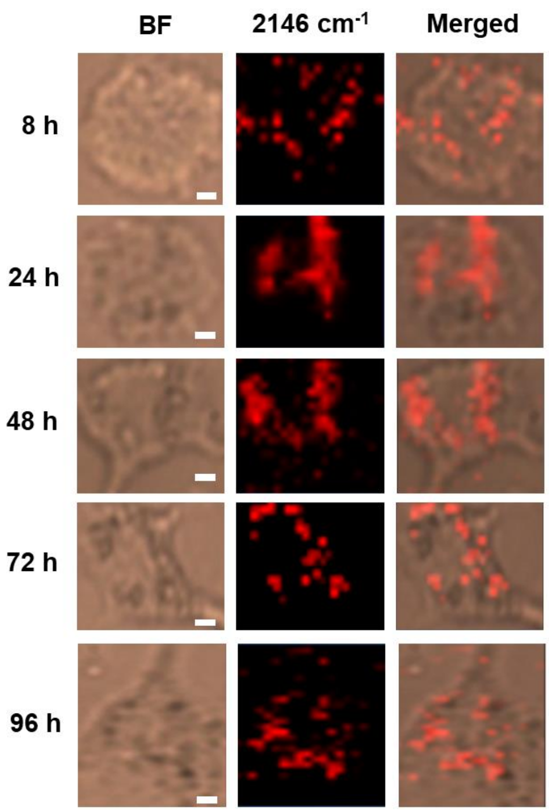

Figure S10. Bright-field (BF), Raman mapping in 2146 cm^{-1} channel, and merged images of BMDCs that treated with APG@OVA NPs for different time, Scale bar, 2 μm.

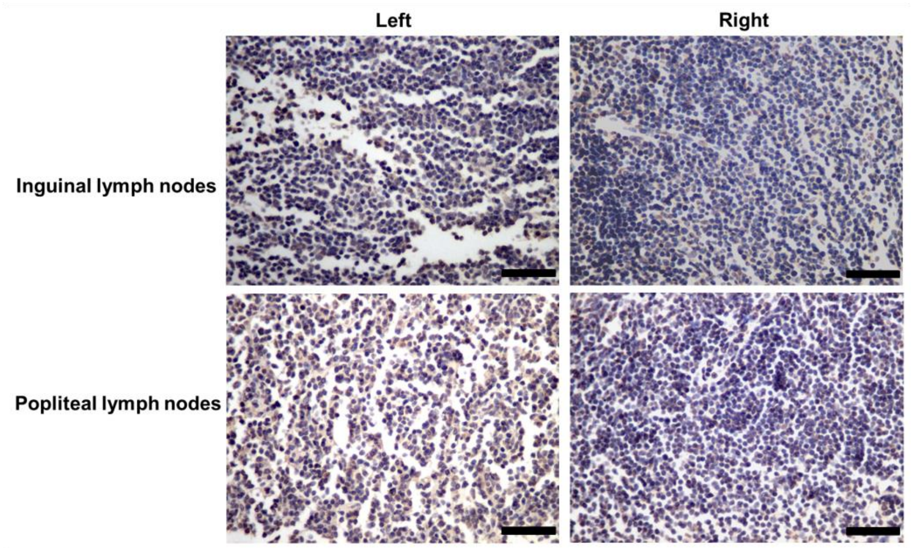

Figure S11. Immunohistochemical staining images of inguinal lymph nodes and popliteal lymph nodes from the mice injected with APG@OVA labeled-BMDCs on the left food pats. Scale bar, 50 μm.

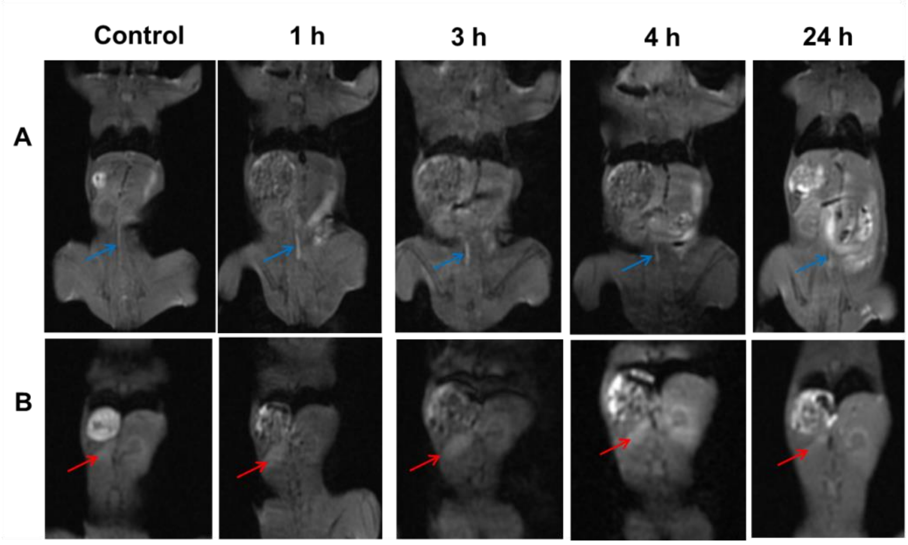

Figure S12. MR imaging of the labeled-BMDCs *in vivo* after intravenous injection of the DCs. The blue arrows indicate vessels (A) and the red arrows indicate spleens (B).
